# Self-efficacy and learning motivation in vocational education: a systematic review

**DOI:** 10.3389/fpsyg.2026.1851592

**Published:** 2026-10-07

**Authors:** Yihong Zhou, Muhd Khaizer Omar, Normala Ismail

**Affiliations:** Faculty of Educational Studies, Universiti Putra Malaysia, Serdang, Selangor, Malaysia

**Keywords:** China, learning motivation, review, self-efficacy, vocational college students

## Abstract

**Background:**

Self-efficacy and learning motivation are widely recognized as key psychological factors influencing students’ engagement and learning outcomes. However, evidence regarding their measurement and relationship among vocational college students remains fragmented.

**Purpose:**

This systematic review synthesizes recent empirical studies on self-efficacy and learning motivation among vocational college students, focusing on research trends, measurement approaches, and reported relationships between the two constructs.

**Methods:**

Following PRISMA-informed procedures, studies published in English-language peer-reviewed journals from January 2022 to early August 2026 were identified through five databases (Web of Science, Scopus, APA PsycINFO, PubMed, and ERIC). After screening and eligibility assessment, 12 studies were included for coding and synthesis. Findings were analyzed descriptively and thematically to summarize study characteristics, measurement instruments, and patterns of association.

**Results:**

The findings showed considerable variation in the measurement of both self-efficacy and learning motivation. Despite this heterogeneity, the included studies generally reported a positive relationship between the two constructs, although the strength and form of the relationship varied across studies and motivational dimensions. Overall, the evidence supports a broadly consistent positive association between self-efficacy and learning motivation among vocational college students, while conceptual and methodological heterogeneity limits direct comparison across studies. Future research should improve construct and measurement consistency and employ longitudinal or experimental designs to clarify the relationship between self-efficacy and learning motivation.

**Systematic review registration:**

CRD420261301263.

## 1 Introduction

Vocational education plays a critical role in modern societies by cultivating technically skilled and practice-oriented talent to support economic development. Over the past decade, China’s vocational education system has trained and supplied an estimated 61 million skilled workers to the labor market, and vocational graduates account for over 70% of newly recruited frontline employees across advanced manufacturing, strategic emerging industries, and modern services (Ministry of Education of the People's Republic of China, 2023). Beyond China, vocational education has been widely recognized as an important driver of economic growth, labor market efficiency, and industrial transformation, with numerous documented benefits across different countries, regions, and sectors (Yan, 2025). Vocational education strengthens employability by developing practical competencies and job-relevant knowledge and has also been linked to greater long-term employment stability; vocational graduates appear to experience more stable employment than their general-education peers prior to age 40 (Woessmann, 2019).

In the Chinese context, vocational education has expanded rapidly and assumed an increasingly important role in workforce preparation. However, vocational college students often study in environments shaped by diverse academic backgrounds, strong employability pressures, and persistent social stereotypes that position vocational pathways as less prestigious than general academic routes. In addition, vocational learning typically combines theoretical study with practical and skills-based training, which places distinctive demands on students’ confidence, persistence, and goal orientation (Hongbiao et al., 2020). These contextual conditions make self-efficacy and learning motivation particularly important for understanding students’ learning experiences in vocational education.

Alongside its economic and social contributions, vocational education has also been shown to exert a significant influence on students’ vocational identity, learning motivation, and self-efficacy. In the context of rapid globalization, technological advancement, and ongoing educational reforms, vocational colleges are increasingly expected to improve teaching quality and foster students’ comprehensive competencies. However, despite expanded access to digital learning resources and instructional technologies, a persistent challenge has emerged: many vocational students exhibit declining learning initiative and reduced engagement, particularly within higher vocational education.

Low learning motivation is often accompanied by diminished self-efficacy, which may give rise to negative emotional experiences such as frustration, boredom, anxiety, and academic burnout (Robinson, 2019; Cheng et al., 2024; Hao et al., 2025). Over time, these psychological challenges can undermine students’ academic engagement, weaken confidence, strain teacher–student relationships, and reduce career preparedness (Aldrup et al., 2018; Szabó, 2018). In response to these concerns, self-efficacy has increasingly been recognized as a vital psychological resource that can help vocational college students cope more effectively with learning-related difficulties and sustain motivation under adverse conditions (Oktarianto et al., 2024; Chen et al., 2025; Liang, 2000).

The purpose of this systematic review is to synthesize recent empirical studies examining self-efficacy and learning motivation among vocational college students. Specifically, the review aims to (a) identify major research trends in studies published from January 2022 to early August 2026, (b) summarize the measurement approaches used to assess self-efficacy and learning motivation, and (c) examine the nature and consistency of the relationship between these two constructs across vocational education contexts. By integrating findings across studies, this review seeks to clarify current evidence and inform future research and educational practice in vocational education.

### 1.1 Related literature

#### 1.1.1 Self-efficacy

Self-efficacy reflects individuals’ confidence in their ability to perform task-related activities successfully (Bandura, 1997; Bandura, 2006). A substantial body of research indicates that self-efficacy is closely associated with students’ learning motivation and may contribute to improved academic achievement (Benawa, 2018). Students with higher self-efficacy are also more likely to accurately identify their strengths and weaknesses and, based on this self-appraisal, set meaningful goals and plan the steps needed to achieve them (Lee et al., 2019). Consistent with this definition, Zagoto (2019) described self-efficacy as individuals’ beliefs in their capability, while Stajkovic and Sergent (2019) further emphasized that such beliefs function as “an important set of proximal determinants of human motivation, affect, and action.” Beyond educational contexts, self-efficacy can enhance individuals’ sense of mastery over occupational tasks, which may in turn strengthen their identification with their job.

#### 1.1.2 Learning motivation

Learning motivation refers to the internal processes that initiate and sustain learners’ engagement in learning activities, including interest, goal orientation, and perceived achievement (Pintrich, 2003). As a multifaceted construct, learning motivation commonly includes intrinsic motivation, extrinsic motivation, and achievement motivation (Ryan and Deci, 2002). Schunk and Zimmerman (2012) similarly conceptualized motivation as a psychological mechanism that initiates, directs, and regulates human action. Extending this view, Gryshchenko et al. (2023) emphasized that motivation shapes the direction, intensity, and persistence of behavior, enabling learners to set higher goals and persist when confronted with challenges. Empirical evidence also suggests that stronger learning motivation is associated with better academic outcomes; for instance, Martínez and Ochoa (2021) reported that motivation can enhance students’ performance, including improvements in oral production.

#### 1.1.3 Relationship between self-efficacy and learning motivation

A growing body of research indicates a positive association between self-efficacy and learning motivation among university students. For instance, Alemayehu and Chen (2023) reported that learning motivation was positively related to learning self-efficacy among Chinese university students. Similarly, Abdolrezapour et al. (2023) and Shane-Simpson et al. (2025) found a positive relationship between self-efficacy and academic motivation among university students in Iran. Consistent with these findings, Chaniago (2024) and Zhou et al. (2026) also reported that accounting students with stronger self-efficacy tended to demonstrate higher academic motivation. Taken together, these studies suggest that students who perceive themselves as capable are generally more willing to engage in learning, persist in the face of difficulty, and respond to academic challenges in a more adaptive manner.

However, this relationship may not be identical across contexts. In vocational education, motivation is often shaped not only by academic goals but also by practical training demands, employability expectations, and students’ perceptions of their future occupational pathways. These features suggest that findings from general university or school populations may not fully capture the dynamics between self-efficacy and learning motivation among vocational college students.

### 1.2 Knowledge of gaps and aims of review

Numerous studies have reported an association between self-efficacy and learning motivation in educational settings; however, evidence relating specifically to vocational college students remains comparatively limited and fragmented. Much of the existing literature has focused on primary, secondary, and general university populations, while vocational education has received relatively less concentrated attention. This imbalance is important because vocational education differs from general academic education in several respects. Students in vocational colleges often learn in contexts shaped by practice-based training, employability expectations, and varied levels of academic preparation, all of which may influence both their sense of competence and their motivation to learn.

In addition, previous studies have conceptualized and measured self-efficacy and learning motivation in different ways, using a wide range of instruments, response formats, and construct dimensions. Such variation makes cross-study comparison difficult and limits the interpretability of findings across contexts. Moreover, although many studies have reported a positive association between the two constructs, the strength and consistency of this relationship appear to vary across educational settings, suggesting that contextual factors may play an important role.

This issue is particularly relevant in the local context, where vocational education has expanded rapidly and plays an increasingly important role in workforce preparation, yet students may still face challenges related to engagement, confidence, and the social perception of vocational pathways. Under such conditions, a context-sensitive synthesis of recent evidence is needed to clarify how self-efficacy and learning motivation have been studied among vocational college students and what patterns have been reported in the literature.

Despite the growing importance of vocational education, there remains a lack of up-to-date systematic literature reviews that synthesize recent findings on this topic. To address this gap, the present review provides a systematic synthesis of empirical studies on self-efficacy and learning motivation among vocational college students, aiming to identify major research trends, summarize measurement approaches used in recent studies, and examine the reported patterns of association between these two constructs. Accordingly, the review addressed the following research questions:

RQ1: What are the characteristics of empirical research on self-efficacy and learning motivation among vocational college students published from January 2022 to early August 2026?

RQ2: How have self-efficacy and learning motivation been conceptualized and measured among vocational college students?

RQ3: What evidence is reported regarding the relationship between self-efficacy and learning motivation among vocational college students?

## 2 Materials and methods

This study employed a systematic literature review (SLR) methodology to ensure transparency and replicability, enabling other researchers to reproduce the review process in future studies. SLRs are valuable for consolidating what is currently known about a topic, as they provide an overarching synthesis of available evidence and can guide future research agendas (Whittemore and Knafl, 2005; Liberati et al., 2009). To address the research question, we systematically searched, screened, and critically appraised the available evidence retrieved from five relevant academic databases.

This systematic review was conducted in accordance with the Preferred Reporting Items for Systematic Reviews and Meta-Analyses (PRISMA) 2020 guidelines. The review protocol was registered in PROSPERO in February 2026 (Registration number: CRD420261301263). The corresponding record has been provided in the reference list for transparency.

### 2.1 Search strategy

Web of Science (WOS), Scopus, APA PsycINFO, ERIC, and PubMed were searched to identify relevant studies. The literature search was limited to studies published in English. The search covered articles published from January 2022, and the final search update was conducted in August 2026. The potential influence of language bias resulting from the English-language restriction was acknowledged.

The search strategy was developed around three key concepts: self-efficacy, learning motivation, and vocational education. Search terms for self-efficacy included “self-efficacy,” “self-efficacy,” “perceived self-efficacy,” “academic self-efficacy,” and “learning self-efficacy.” Terms related to learning motivation included “learning motivation,” “motivation to learn,” and “academic motivation.” The vocational education search terms included “higher vocational education,” “higher vocational college*,” “vocational college*,” “post-secondary vocational education,” “postsecondary vocational education,” “polytechnic*,” “technical college*,” “vocational education and training,” “vocational education,” “TVET,” and “VET.” Terms within each concept were combined using the Boolean operator “OR,” while the three concept groups were combined using “AND.” The key search concepts and terms are presented in Table 1.

**Table 1:** Search strings.

Search concept	Search terms (Title/Abstract/Keywords)
self-efficacy	(“self-efficacy” OR “self efficacy” OR “perceived self-efficacy” OR “academic self-efficacy” OR “learning self-efficacy”)
Learning Motivation	(“learning motivation” OR “motivation to learn” OR “academic motivation”)
Vocational education	(“higher vocational education” OR “higher vocational college*” OR “vocational college*” OR “post-secondary vocational education” OR “postsecondary vocational education” OR polytechnic* OR “technical college*” OR “vocational education and training” OR “vocational education” OR TVET OR VET)
Final search string	(“self-efficacy” OR “self efficacy” OR “perceived self-efficacy” OR “academic self-efficacy” OR “learning self-efficacy”) AND (“learning motivation” OR “motivation to learn” OR “academic motivation”) AND (“higher vocational education” OR “higher vocational college*” OR “vocational college*” OR “post-secondary vocational education” OR “postsecondary vocational education” OR polytechnic* OR “technical college*” OR “vocational education and training” OR “vocational education” OR TVET OR VET)

Databases: Web of Science, Scopus, APA PsycINFO, ERIC, PubMed.

Publication Dates: January 2022 to early August 2026.

The same core search strategy was applied across all five databases, with adjustments made to accommodate database-specific search fields and syntax. The search strings were developed with assistance from a subject librarian. The database searches identified a total of 66 records: 26 from Web of Science, 16 from Scopus, 14 from PubMed, 6 from APA PsycINFO, and 4 from ERIC. The complete database-specific search strategies and corresponding search results are presented in Table 2.

**Table 2:** Database search strategies and search results.

Database	Strategy	Results
Web of Science	“self-efficacy” OR “self efficacy” OR “perceived self-efficacy” OR “academic self-efficacy” OR “learning self-efficacy” (All Fields) and “learning motivation” OR “motivation to learn” OR “academic motivation” (All Fields) and “higher vocational education” OR “higher vocational college*” OR “vocational college*” OR “post-secondary vocational education” OR “postsecondary vocational education” OR polytechnic* OR “technical college*” OR “vocational education and training” OR “vocational education” OR TVET OR VET (All Fields)	26
Scopus	TITLE-ABS-KEY (“self-efficacy” OR “self efficacy” OR “perceived self-efficacy” OR “academic self-efficacy” OR “learning self-efficacy”) AND TITLE-ABS-KEY (“learning motivation” OR “motivation to learn” OR “academic motivation”) AND TITLE-ABS-KEY (“higher vocational education” OR “higher vocational college*” OR “vocational college*” OR “post-secondary vocational education” OR “postsecondary vocational education” OR polytechnic* OR “technical college*” OR “vocational education and training” OR “vocational education” OR TVET OR VET)	16
PubMed	((“self-efficacy” OR “self efficacy” OR “perceived self-efficacy” OR “academic self-efficacy” OR “learning self-efficacy”) AND (“learning motivation” OR “motivation to learn” OR “academic motivation”)) AND (“higher vocational education” OR “higher vocational college*” OR “vocational college*” OR “post-secondary vocational education” OR “postsecondary vocational education” OR polytechnic* OR “technical college*” OR “vocational education and training” OR “vocational education” OR TVET OR VET)	14
APA PsyINFO	noft((“self-efficacy” OR “self efficacy” OR “perceived self-efficacy” OR “academic self-efficacy” OR “learning self-efficacy”)) AND noft((“learning motivation” OR “motivation to learn” OR “academic motivation”)) AND noft((“higher vocational education” OR “higher vocational college*” OR “vocational college*” OR “post-secondary vocational education” OR “postsecondary vocational education” OR polytechnic* OR “technical college*” OR “vocational education and training” OR “vocational education” OR TVET OR VET))	6
ERIC	(“self-efficacy” OR “self efficacy” OR “perceived self-efficacy” OR “academic self-efficacy” OR “learning self-efficacy”) AND (“learning motivation” OR “motivation to learn” OR “academic motivation”) AND (“higher vocational education” OR “higher vocational college*” OR “vocational college*” OR “post-secondary vocational education” OR “postsecondary vocational education” OR polytechnic* OR “technical college*” OR “vocational education and training” OR “vocational education” OR TVET OR VET)	4
Total		66

### 2.2 Inclusion\exclusion criteria for studies

This review applied two sets of eligibility criteria—inclusion and exclusion—to determine which studies were eligible for analysis. To structure and operationalize these criteria, we drew on two established frameworks: PEO (Population, Exposure, Outcome) and SPIDER (Sample, Phenomenon of Interest, Design, Evaluation, Research type) (Cooke et al., 2012). These frameworks helped translate the scope of the review into clear screening rules and supported a comprehensive and systematic identification of relevant empirical studies. The full inclusion and exclusion criteria used in the screening process are presented in Table 3.

**Table 3:** Eligibility criteria for study selection.

Criterion	Inclusion criteria	Exclusion criteria
Population	Students enrolled in post-secondary or higher vocational education, including vocational colleges and vocational universities.	Students in general universities; primary or secondary school students; upper secondary vocational students.
Phenomenon/exposure	Studies measuring both self-efficacy and learning motivation.	Studies examining only one of the two constructs or neither construct.
Country setting	Any country	None
Outcome	Studies reporting data on the relationship between self-efficacy and learning motivation	Studies that did not report the relationship between the two constructs.
Language	Full-text articles published in English.	Non-English publications.
Publication period	From January 2022 to early August 2026	Studies published outside the specified period.
Study design	Empirical quantitative, qualitative, or mixed-methods studies.	Reviews, theoretical papers, editorials, conference papers, book chapters, dissertations, and studies without original data.

Studies were selected according to predefined inclusion and exclusion criteria. Eligible studies focused on students enrolled in post-secondary or higher vocational education, including vocational colleges and vocational universities. Studies involving general university students, primary or secondary school students, or upper secondary vocational students were excluded. Studies were required to examine both self-efficacy and learning motivation and to report findings on the relationship between the two constructs. Studies examining only one of these constructs, or those that did not provide sufficient information on their relationship, were excluded. Studies conducted in any country or region were considered. Only full-text articles published in English were included. Regarding study design, empirical quantitative, qualitative, and mixed-methods studies reporting original data were eligible, whereas reviews, theoretical or conceptual papers, editorials, conference papers, book chapters, dissertations or theses, and other publications without original empirical data were excluded. The review was limited to studies published from January 2022 to early August 2026. This period was selected to capture recent research on self-efficacy and learning motivation in vocational education and to reflect current educational contexts.

### 2.3 Data extraction

The study selection process followed the Preferred Reporting Items for Systematic Reviews and Meta-Analyses (PRISMA) guidelines. During the identification stage, searches across five databases yielded 66 records: Web of Science (*n* = 26), Scopus (*n* = 16), PubMed (*n* = 14), APA PsycINFO (*n* = 6), and ERIC (*n* = 4). After removing 23 duplicate records, 43 records remained for title and abstract screening. Two conference papers were excluded during this stage because they did not meet the predefined eligibility criteria, leaving 41 reports for retrieval. All 41 reports were successfully retrieved and assessed for eligibility. Following the full-text assessment, 29 reports were excluded. Of these, 22 did not measure both self-efficacy and learning motivation, four did not report the relationship between the two constructs, two involved ineligible populations, and one was a review article. Consequently, 12 studies met the eligibility criteria and were included in the final review for data extraction and synthesis. The study selection process is summarized in the PRISMA flow diagram (Figure 1). References were managed using EndNote X9, while Microsoft Excel and Word were used to support data management, extraction, and reporting.

**Figure 1:**
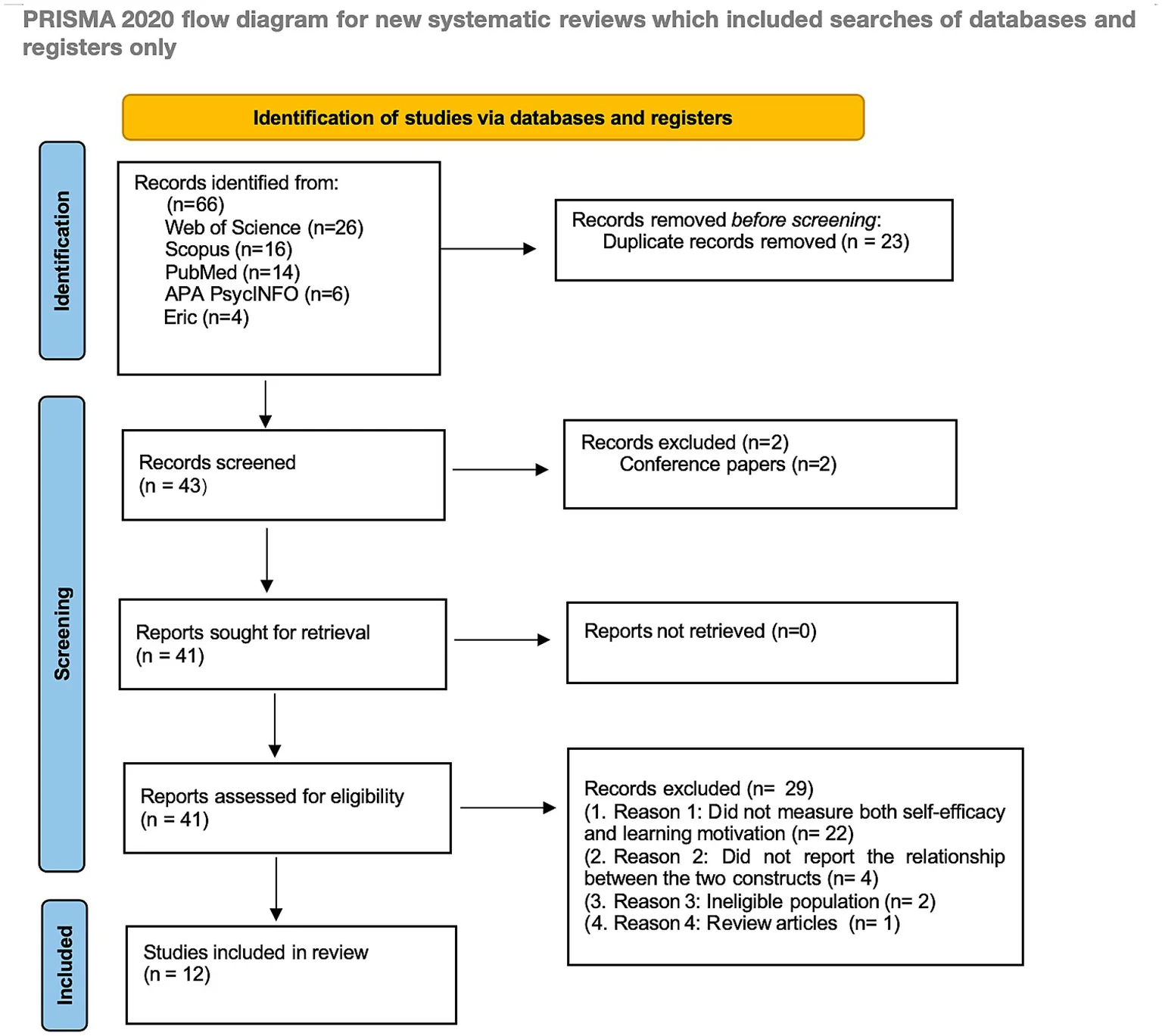
PRISMA flow diagram.

### 2.4 Quality assessment of included studies

The methodological quality of the included studies was assessed using the Crowe Critical Appraisal Tool (CCAT), version 1.4 (Crowe, 2013). The CCAT comprises eight appraisal categories: Introduction, Background, Methods, Sampling, Data Collection, Ethical Matters, Results, and Discussion. Each domain was rated on a scale from 0 to 5, with higher scores indicating stronger methodological quality and reporting. The domain-level scores and total scores for each included study are presented in Appendix A (Table A2; Supplementary Materials). Two reviewers independently assessed each study, and discrepancies in ratings were resolved through discussion and consensus. Methodological quality was not used as an additional criterion for study exclusion; rather, the appraisal findings were considered when interpreting the strength and consistency of the evidence.

### 2.5 Data extraction and management

A structured data extraction process was used to organize the evidence from the included studies. For each article, key bibliographic and methodological information was recorded, including author(s), publication year, country/region, title, research purpose, study design, sample characteristics (participants and context), measurement instruments, and main findings. Where applicable, variables were also coded according to their analytical roles, such as predictors, outcomes, mediators, and moderators. These data were entered into a predefined coding framework in Microsoft Excel to support consistency, transparency, and cross-study comparison.

Data extraction was conducted independently by the first two authors using the same coding framework. After the initial extraction, the coded information was compared across reviewers. Any discrepancies in coding or interpretation were resolved through discussion, and a third author was consulted when necessary. References were managed using EndNote X9, while Microsoft Excel was used for coding and organizing extracted data, and Microsoft Word was used for reporting and documentation.

### 2.6 Synthesis and analysis of results

The findings were synthesized using a narrative synthesis approach to identify patterns across the included quantitative studies. Data extracted from each study included study characteristics, participant characteristics, self-efficacy measures, learning motivation measures, statistical methods, effect estimates, and the reported relationship between the two constructs. Studies were then compared systematically according to the review objectives rather than pooled statistically because of substantial heterogeneity in measurement instruments, learning contexts, and analytical approaches.

The synthesis was organized into three analytical categories: (1) research characteristics and trends, including publication year and geographical distribution; (2) measurement approaches, examining how self-efficacy and learning motivation were conceptualized and operationalized across studies; and (3) the relationship between self-efficacy and learning motivation, summarizing reported effect estimates, analytical models, and methodological differences. Within the third category, different forms of self-efficacy (e.g., general, academic, English-learning, digital, and emotional self-efficacy) and learning motivation (e.g., intrinsic, extrinsic, achievement, deep, and surface motivation) were synthesized separately where possible to improve conceptual clarity. Methodological quality was considered when interpreting the consistency and strength of the evidence.

## 3 Results

### 3.1 Characteristics of the included studies

As shown in Figure 2, the final sample comprised 12 studies published between 2022 and 2026. Publication output was highest in 2026 (*n* = 5), followed by 2024 (*n* = 4), 2025 (*n* = 2), and 2022 (*n* = 1). No eligible studies published in 2023 were identified. All 12 included studies employed quantitative research designs, indicating a clear methodological concentration within the existing evidence base. Although the number of studies varied across the review period, the relatively high number of publications in 2024 and 2026 suggests increasing research attention to self-efficacy and learning motivation in vocational education in recent years.

**Figure 2:**
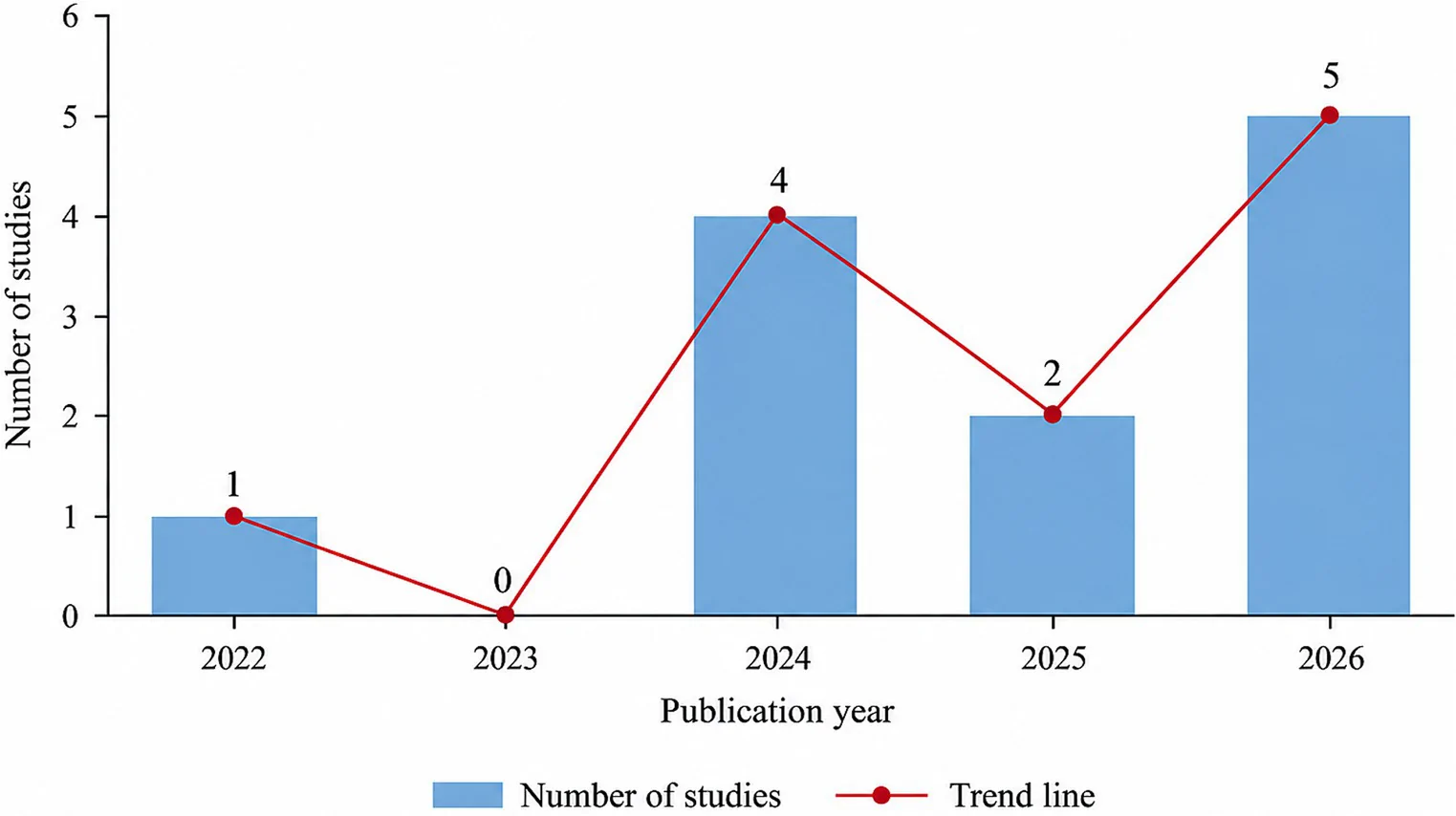
Distribution of included studies by publication year (*N* = 12).

As shown in Figure 3, the included studies were conducted in three countries, with China accounting for the majority (*n* = 10, 83.3%), while Malaysia and Indonesia each contributed one study (*n* = 1, 8.3%). A further examination of the Chinese studies showed that they covered eight provinces or provincial-level regions (Figure 4). Sichuan had the highest number of studies (*n* = 4, 40.0%), whereas Hunan, Xinjiang, Anhui, Guangdong, Shandong, Zhejiang, and Jiangsu each had one study (*n* = 1, 10.0%). These findings show that, although the Chinese studies represented several geographical areas, the evidence was unevenly distributed and was particularly concentrated in Sichuan.

**Figure 3:**
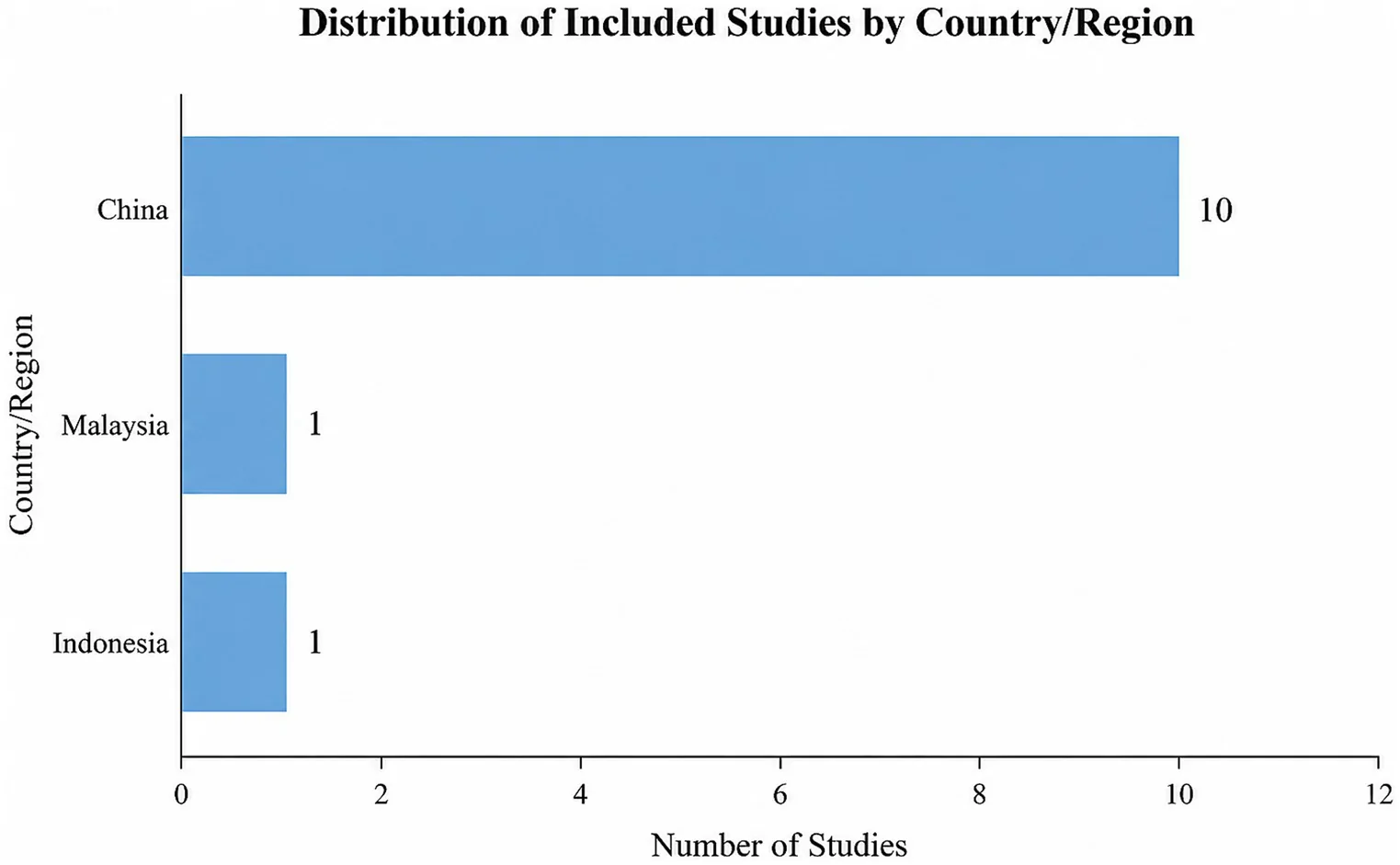
Distribution of included studies by country/region (*N* = 12).

**Figure 4:**
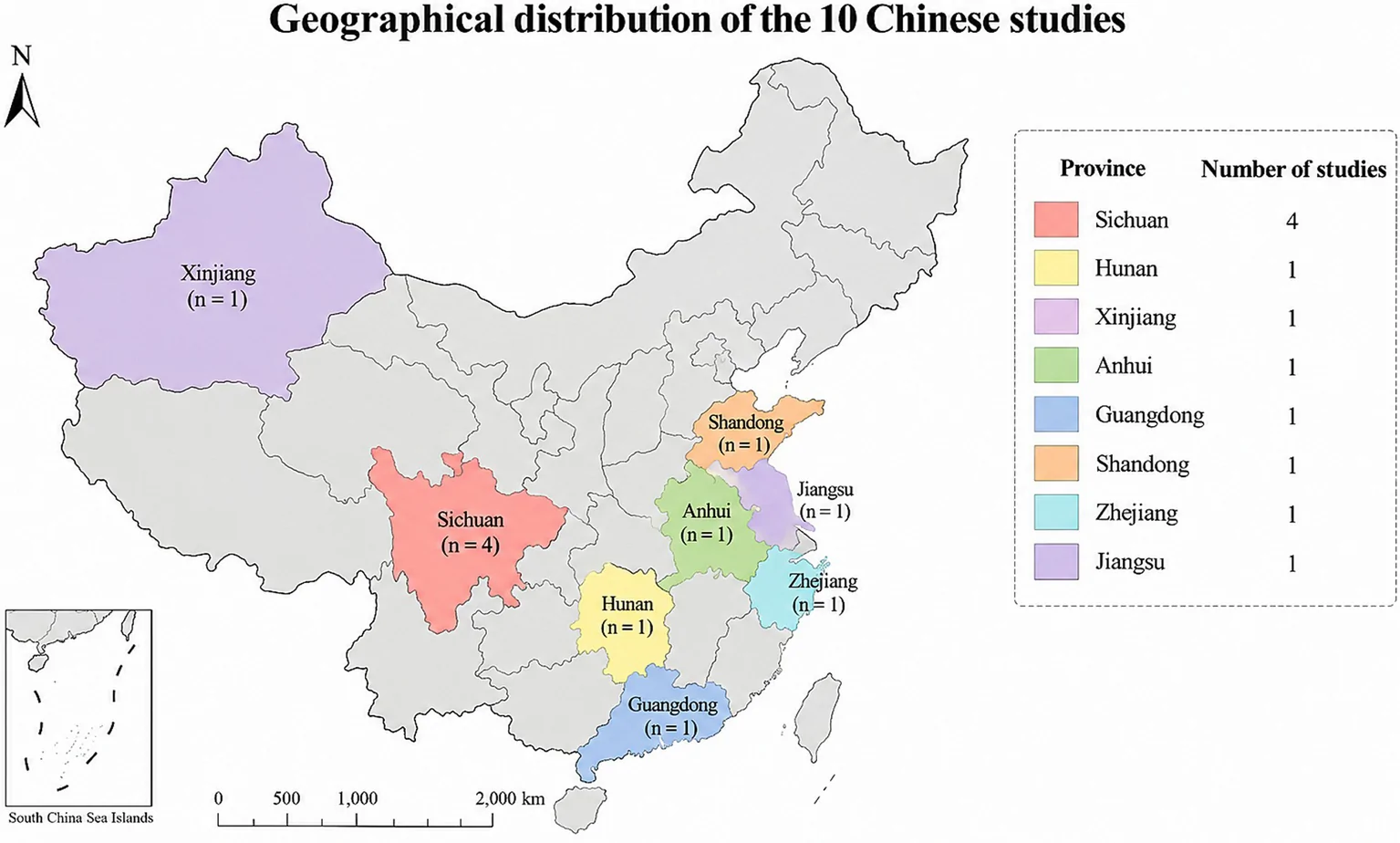
Geographical distribution of the included Chinese studies by province (*n* = 10).

Figure 5 presents the keyword co-occurrence network based on the 12 studies that provided author keywords. Self-efficacy and learning motivation were the most prominent concepts in the network and were closely connected with a range of related keywords. Different forms of self-efficacy were represented, including academic, learning, digital, regulatory emotional, and English-speaking self-efficacy. Other keywords reflected a variety of research contexts and related factors, such as vocational education, digital learning, academic motivation, psychological well-being, learning outcomes, and student characteristics. These patterns suggest that the relationship between self-efficacy and learning motivation has been explored from multiple perspectives, although the diversity of keywords also indicates considerable variation in how the two constructs have been studied.

**Figure 5:**
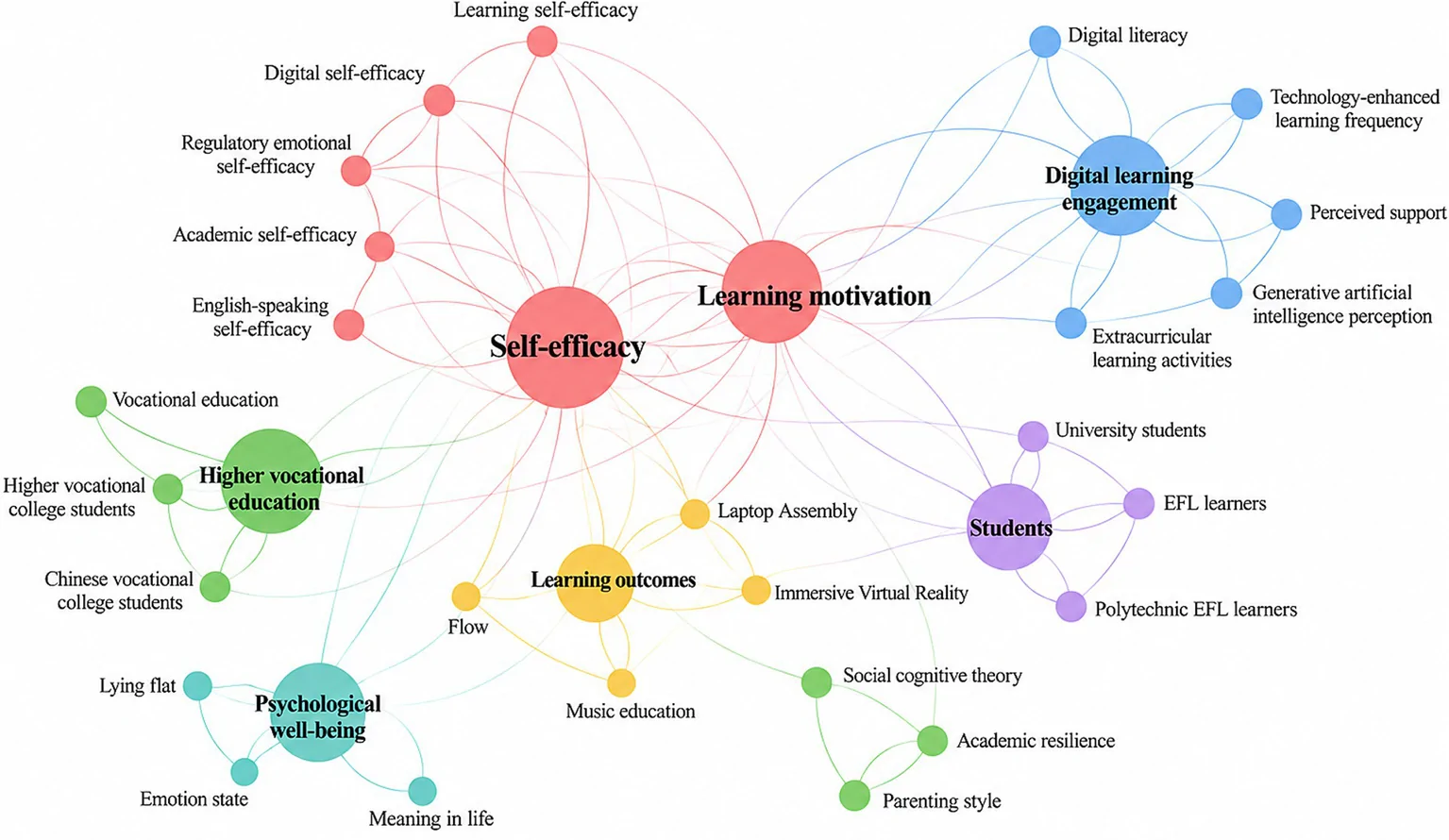
Keyword co-occurrence network of the included studies with available author keywords (*n* = 12).

### 3.2 Thematic findings addressing the research questions

#### 3.2.1 Measurements of self-efficacy

Considerable variation was found in how self-efficacy was operationalized across the included studies. Chen et al. (2026) and Sindu and Kertiasih (2024) adopted similar approaches, with both measuring self-efficacy using items based on Bandura (1997) and Zimmerman (2000). Both studies employed a seven-point Likert scale and focused on students’ confidence in performing academic or learning-related tasks. In contrast, Liu et al. (2026) and Su et al. (2024) used the General Self-Efficacy Scale (GSE; Schwarzer, 1997), which assesses broader beliefs about one’s ability to deal with difficulties and achieve goals. Shengyao et al. (2024) similarly used a relatively general measure of self-efficacy developed by Rowbotham and Schmitz.

Other studies conceptualized self-efficacy in more specific learning domains. Shi et al. (2026) used the Chinese version of the Academic Self-Efficacy Scale (Pintrich and De Groot, 1990; revised by Liang Y., 2000), whereas Tang and Osman (2022) employed the Learning Self-Efficacy Scale (LSS; Liang Y., 2000), which includes behavioral and ability-related learning self-efficacy. Two studies focused specifically on English learning. Lin et al. (2025) used the English-Speaking Self-Efficacy Scale (Chen, 2009), covering confidence in achieving speaking goals, completing speaking tasks, and overcoming difficulties, while Zhang (2024) used a modified English Learning Self-Efficacy Scale. Self-efficacy was further contextualized in technology-related learning: Dong (2025) employed the Digital Self-Efficacy Scale (DSES; Ulfert-Blank and Schmidt, 2022), which assesses several aspects of digital competence, while Li et al. (2026) used an adapted Computer Self-Efficacy Scale (Compeau and Higgins, 1995). Liang and Fan (2026) took a different approach by using the Chinese version of the Regulatory Emotional Self-Efficacy Scale (RESE; Caprara et al., 2008), covering the expression of positive emotions and the management of negative emotional experiences.

Overall, the measures indicate substantial heterogeneity in the conceptualization of self-efficacy. Although all studies addressed beliefs about personal capability, the object of these beliefs differed considerably, ranging from general capability beliefs to academic, learning, English-speaking, digital, computer-related, and emotion-regulation capabilities. Most studies used five-point Likert scales, whereas Chen et al. (2026) and Sindu and Kertiasih (2024) used seven-point response formats. More importantly, the use of both general and highly domain-specific measures means that the studies did not assess an entirely equivalent construct. For example, confidence in managing emotions or performing digital tasks may not represent the same type of efficacy belief as confidence in completing academic tasks or learning English. This measurement diversity broadens the contexts in which self-efficacy has been examined in vocational education, but it also limits direct comparability across studies and should be considered when interpreting differences in the reported relationships between self-efficacy and learning motivation.

#### 3.2.2 Measurements of learning motivation

The included studies also showed considerable variation in the measurement of learning motivation. Some studies adopted theory-based or established academic motivation measures. Chen et al. (2026) assessed learning motivation using items based on Deci and Ryan (2000) and Ryan and Deci (2020), focusing on effort, persistence, and the desire to improve academic performance. Li et al. (2026) used an adapted Short Academic Motivation Scale (Kotera et al., 2023), which covered seven types of motivation, including amotivation, external regulation, introjected regulation, identified regulation, and three forms of intrinsic motivation. Su et al. (2024) used five items adapted from the Academic Motivation Scale (AMS; Vallerand et al., 1992), while Shi et al. (2026) assessed academic motivation using a 20-item scale comprising deep and surface motivation. These studies generally conceptualized motivation in terms of students’ reasons for engaging in learning, but they differed in the extent to which intrinsic, extrinsic, and regulatory forms of motivation were distinguished.

Other studies used measures tailored to specific learning contexts or broader motivational dimensions. Dong (2025) employed the Online Learning Motivation Scale (OLMS; Fowler, 2018), which included intrinsic and extrinsic goal orientation, control of learning beliefs, self-efficacy, task value, social engagement, and instructor support. Sindu and Kertiasih (2024) used a modified Motivated Strategies for Learning Questionnaire (MSLQ) for an immersive VR learning context. Lin et al. (2025) used the English Learning Motivation Scale (Gao et al., 2003), whereas Zhang (2024) employed a learning motivation measure adapted from the Learning Process Questionnaire, including extrinsic, deep, and achievement motivation. Liang and Fan (2026) used a six-dimensional Learning Motivation Scale covering intellectual pursuit, social orientation, material pursuit, fear of failure, personal achievement, and small-group orientation. Tang and Osman (2022) used the Learning Motivation Scale (Tian and Pan, 2006), which assessed interest in knowledge, ability pursuit, altruism orientation, and reputation acquisition. Shengyao et al. (2024) assessed academic motivation through intrinsic and extrinsic dimensions, while Liu et al. (2026) used the Revised Learning Process Questionnaire (Kember et al., 2004), distinguishing deep and surface motives. Most studies used five-point Likert response formats, although some used other formats, such as the nine-point scale reported by Zhang (2024).

Overall, learning motivation was not operationalized as a uniform construct across the included studies. Some measures focused mainly on intrinsic and extrinsic motivation, whereas others emphasized deep and surface motives, achievement-related goals, regulatory styles, perceived task value, social engagement, or context-specific reasons for learning. In particular, several instruments incorporated constructs that extend beyond motivation in a narrow sense. For example, the OLMS used by Dong (2025) included self-efficacy, instructor support, and social engagement alongside motivational components. This broader operationalization may blur the conceptual boundary between learning motivation and related psychological or contextual factors. Consequently, although all studies examined “learning motivation,” the content captured by the measures differed substantially, limiting direct comparability across studies and making it important to interpret reported associations with self-efficacy in relation to the specific motivational construct assessed.

#### 3.2.3 Relationship between learning motivation and self-efficacy

Across the 12 included studies, the evidence generally indicated a positive relationship between self-efficacy and learning motivation among students in vocational education. Five studies reported correlation coefficients and consistently identified significant positive associations between the two constructs. Chen et al. (2026) found a positive correlation between self-efficacy and learning motivation (*r* = 0.290, *p* < 0.001), while Liu et al. (2026) reported a somewhat higher correlation (*r* = 0.440, *p* < 0.001). Similarly, Shi et al. (2026) found that academic self-efficacy was positively correlated with academic motivation (*r* = 0.486, *p* < 0.01). Liang and Fan (2026) reported a positive correlation between regulatory emotional self-efficacy and learning motivation (*r* = 0.541, *p* < 0.001), and Tang and Osman (2022) found the largest correlation among these studies between learning self-efficacy and learning motivation (*r* = 0.712, *p* < 0.01). Taken together, the correlation coefficients ranged from *r* = 0.290 to 0.712, indicating approximately moderate to strong positive associations. However, these coefficients should be interpreted in relation to the specific constructs measured, as the studies examined different forms of self-efficacy and learning motivation rather than identical constructs.

Studies using regression or structural models showed a similar overall pattern. Dong (2025) identified a significant positive relationship between digital self-efficacy and online learning motivation (*β* = 0.432, *p* < 0.05), while Li et al. (2026) found that computer self-efficacy positively predicted learning motivation (*β* = 0.278, *p* < 0.001). In language-learning contexts, Lin et al. (2025) reported that English-speaking self-efficacy positively predicted English learning motivation (*β* = 0.240, *p* < 0.001). Significant positive relationships were also reported by Shengyao et al. (2024) for self-efficacy and academic motivation (*β* = 0.642, *p* < 0.001), Sindu and Kertiasih (2024) for self-efficacy and learning motivation in an immersive VR learning context (*β* = 0.428, *p* < 0.001), and Su et al. (2024) for self-efficacy and learning motivation (*β* = 0.176, *p* = 0.004). Although the standardized coefficients varied across studies, they consistently indicated positive relationships. Direct comparison of the β coefficients should nevertheless be avoided because the studies differed in their measures, model specifications, learning contexts, and other variables included in the statistical models.

One study provided a more differentiated picture of the relationship. Zhang (2024) examined English learning self-efficacy in relation to different dimensions of learning motivation and found significant positive relationships with intrinsic motivation (*β* = 0.573, *p* < 0.001) and achievement motivation (*β* = 0.491, *p* < 0.05), but not with extrinsic motivation (*β* = 0.054, *p* > 0.05). This finding suggests that the positive relationship between self-efficacy and learning motivation may not be equally evident across all motivational dimensions. Overall, the evidence across the included studies supports a broadly consistent positive association between self-efficacy and learning motivation in vocational education, but the magnitude and pattern of this association varied according to how the two constructs were operationalized. The findings therefore point to consistency in the direction of the relationship but heterogeneity in its strength and form. Given the variation in measurement instruments and statistical approaches, the reported coefficients should not be treated as directly comparable effect sizes. Rather, the evidence is more appropriately interpreted as showing a general positive pattern across different vocational learning contexts, with some variation according to the specific type of self-efficacy and motivation examined.

## 4 Discussion

Based on the synthesis of 12 empirical studies, this systematic review identified two main patterns in the literature on self-efficacy and learning motivation among vocational college students. First, considerable variation was evident in how both constructs were conceptualized and measured across studies. Second, despite this measurement heterogeneity, the evidence generally showed a consistent positive relationship between self-efficacy and learning motivation, although the magnitude and form of this relationship varied across studies and motivational dimensions. These findings highlight both the consistency of the overall relationship and the conceptual and methodological diversity of the existing evidence base.

The findings of this review highlight considerable heterogeneity in the conceptualization and measurement of self-efficacy in vocational education. No single measurement approach predominated across the included studies. Instead, self-efficacy was examined at different levels of specificity, ranging from general beliefs about personal capability to academic, learning, English-related, digital, computer-related, and emotion-regulation self-efficacy. This diversity may partly reflect the heterogeneous nature of vocational education, in which students engage in different academic, practical, language-related, and technology-supported learning activities. The use of domain-specific measures is therefore not necessarily a methodological weakness, as such measures may better capture students’ confidence in performing particular tasks. Nevertheless, general and domain-specific self-efficacy measures should not be treated as interchangeable. A broad belief in one’s ability to cope with difficulties is conceptually different from confidence in performing a specific activity, such as speaking English or using digital technologies. The lack of a dominant measurement approach therefore makes it important to consider the level of specificity of self-efficacy when comparing findings across studies.

A similar, and in some respects greater, conceptual diversity was evident in the measurement of learning motivation. The included studies operationalized motivation through intrinsic and extrinsic motivation, deep and surface motives, achievement-related motivation, regulatory styles, and other context-specific motivational dimensions. This variation indicates that “learning motivation” was used as an umbrella term for constructs that were related but not necessarily equivalent. Of particular concern is that some measures extended beyond motivation in a narrow sense. For example, the Online Learning Motivation Scale used by Dong (2025) incorporated self-efficacy, social engagement, and instructor support alongside motivational components. This creates a potential problem of construct overlap because self-efficacy was simultaneously treated as a separate variable in examining its relationship with learning motivation. In such cases, part of the observed relationship may reflect overlapping measurement content rather than an association between two fully distinct constructs. This does not invalidate the findings, but it suggests that they should be interpreted more cautiously and that clearer conceptual boundaries between self-efficacy, motivation, and related contextual factors are needed.

Despite these differences in conceptualization and measurement, one of the clearest findings of the review was the consistency in the direction of the relationship between self-efficacy and learning motivation. All five studies reporting bivariate correlations identified significant positive associations, with coefficients ranging from approximately moderate to strong (*r* = 0.290–0.712). Studies using regression or structural models similarly reported significant positive relationships in most cases. The consistency of this pattern across general, academic, language-related, digital, and other learning contexts suggests that students who hold stronger beliefs in their capabilities also tend to report higher levels of learning motivation. This pattern is theoretically plausible because confidence in one’s capability to perform learning tasks may be associated with greater willingness to invest effort, persist when difficulties arise, and engage with learning activities. This finding is consistent with social cognitive theory, which proposes that efficacy beliefs influence individuals’ motivation, behavioral choices, and persistence in the face of difficulties. In vocational education, where learning is often task-oriented and closely linked to practical skill development, students with higher self-efficacy may be more confident in completing authentic learning tasks and therefore demonstrate stronger motivation to participate in classroom and workplace-related learning activities. These findings suggest that self-efficacy may be an important factor to consider in understanding and supporting learning motivation in vocational education (Xu et al., 2018; Gao, 2021; In'am and Sutrisno, 2021; Dadandı, 2023; Alemayehu and Chen, 2023; Jia and Tu, 2024).

Nevertheless, the consistency of the positive association should not be interpreted as evidence of a causal relationship. The included literature was dominated by quantitative observational research, making it difficult to establish the temporal ordering of self-efficacy and learning motivation. Higher self-efficacy may contribute to greater motivation, but greater motivation and successful learning experiences may also strengthen students’ efficacy beliefs over time. A reciprocal relationship is therefore possible, although the available evidence does not allow this possibility to be established. Moreover, evidence from previous research suggests that the relationship may be sensitive to learning conditions. For example, Yong and Thi (2022) reported only a weak relationship between self-efficacy and learning motivation during emergency online learning. Although this study falls outside the evidence synthesized in the present review, it provides a useful contrast to the predominantly positive pattern observed here. Under disrupted learning conditions, constraints such as unstable internet access, inadequate digital infrastructure, limited interaction with teachers and peers, and reduced institutional support may restrict students’ opportunities to translate confidence in their capabilities into sustained motivation. This suggests that self-efficacy should not be viewed as operating independently of the learning environment; rather, its association with motivation may be strengthened or constrained by the conditions in which learning occurs.

At the same time, the relationship was not completely uniform. Zhang (2024), for example, found that English learning self-efficacy was positively related to intrinsic and achievement motivation but not significantly related to extrinsic motivation. This finding is important because it suggests that the relationship may depend on the type of motivation being examined. Confidence in one’s learning capabilities may be more closely connected with motivation arising from interest, competence, or achievement than with motivation driven primarily by external incentives or pressures. More broadly, differences in the magnitude of the reported relationships across the included studies should be interpreted cautiously. The studies differed not only in populations and learning contexts but also in the specificity of self-efficacy measures, definitions of learning motivation, scale content, and statistical methods. Moreover, correlation coefficients and standardized regression or path coefficients represent different statistical estimates and should not be directly compared as equivalent effect sizes. Thus, the evidence is more appropriately characterized as showing consistency in the direction of the relationship but heterogeneity in its strength and form, rather than demonstrating a single uniform magnitude of association across vocational education.

The findings of this review also have several practical implications for teachers, vocational institutions, and educational policymakers. For teachers, instructional practices that provide timely feedback, appropriate scaffolding, and opportunities for successful task completion may help strengthen students’ self-efficacy and sustain their learning motivation. Teachers may also encourage students to set achievable learning goals and reflect on their learning progress to foster confidence and persistence. At the institutional level, vocational colleges should create supportive learning environments by providing adequate learning resources, digital infrastructure, and professional development opportunities for teachers. Institutions may also integrate psychological support and learning guidance into student support services to promote both academic confidence and motivation. At the policy level, educational policymakers should consider incorporating the development of students’ self-efficacy and learning motivation into vocational education policies, curriculum frameworks, and quality evaluation systems. Policies that encourage evidence-based teaching practices, equitable access to learning resources, and teacher training programmers may contribute to improving students’ learning engagement and educational outcomes.

This study has several limitations. Firstly, the reviewed evidence relies largely on cross-sectional designs, which restricts causal interpretation. Secondly, variations in measurement instruments and learning contexts limit direct comparability across studies. Third, only 12 studies met the predefined eligibility criteria despite systematic searches across five major databases. This number reflects the actual body of eligible evidence identified through the predefined search and selection procedures rather than an intentionally restricted sample, but the relatively small evidence base nevertheless limits the breadth and generalizability of the findings. In addition, the inclusion of English-language publications only may have introduced language bias and excluded potentially relevant studies. Future research should adopt longitudinal or experimental designs to examine the relationship between self-efficacy and learning motivation and explore contextual moderators such as learning environment quality and technological readiness. In addition, greater measurement transparency, more consistent construct definitions, and broader search strategies incorporating internationally recognized terminology would improve the comprehensiveness and comparability of future evidence syntheses.

## 5 Conclusion

This systematic review synthesized 12 empirical studies examining self-efficacy and learning motivation among vocational college students, with particular attention to research characteristics, measurement approaches, and the relationship between the two constructs. Overall, the findings showed considerable heterogeneity in how both self-efficacy and learning motivation were conceptualized and measured. Self-efficacy ranged from general capability beliefs to academic, learning, language-related, digital, computer-related, and emotion-regulation forms, while learning motivation was operationalized through a variety of dimensions, including intrinsic and extrinsic motivation, deep and surface motives, and achievement-related motivation. Despite this measurement diversity, the evidence showed a broadly consistent positive association between self-efficacy and learning motivation. However, the strength and form of the relationship varied across studies, and one study showed different relationships across specific motivational dimensions. These findings suggest that the relationship is better understood as generally positive but heterogeneous rather than as a single uniform association across vocational education contexts.

The review highlights the importance of considering both construct specificity and educational context when examining self-efficacy and learning motivation in vocational education. The diversity of measures suggests a need for clearer conceptual definitions and better alignment between the forms of self-efficacy and motivation being assessed, while still recognizing the varied learning environments and tasks that characterize vocational education. The findings also indicate that self-efficacy may be an important factor to consider when supporting students’ learning motivation, although the predominantly observational evidence does not permit causal conclusions. Future research should employ longitudinal and experimental designs, examine a wider range of vocational education systems and learning contexts, and improve conceptual and measurement consistency. A broader and methodologically stronger evidence base is needed to clarify how and under what conditions self-efficacy and learning motivation are related among vocational students.

## Data Availability

The datasets presented in this study can be found in online repositories. The names of the repository/repositories and accession number(s) can be found in the article/supplementary material.
