# Supplementary material for: Self-efficacy and learning motivation in vocational education: a systematic review
Source: Front Psychol. 2026 Oct 7;17:1851592. doi: 10.3389/fpsyg.2026.1851592 (PMC13683600; doi:10.3389/fpsyg.2026.1851592)
Supplement: Supplementary file 1 [file Supplementary_file_1.docx]

Appendix A

Table A1. Characteristics and measurement approaches of the included studies (n = 12)

| **No.** | **Authors** | **Country (Province/Region)** | **Participants (N)** | **Design** | **Self-Efficacy Measure** | **Learning Motivation Measure** | **Main findings** |
| --- | --- | --- | --- | --- | --- | --- | --- |
| 1 | Chen et al., (2026) | China  （Xinjiang） | 420 | Quantitative | Self-efficacy items based on Bandura (1997) and Zimmerman (2000). | Learning motivation items based on Deci and Ryan (2000) and Ryan and Deci (2020). | Positive correlation between self-efficacy and learning motivation (r = .290, p < .001). |
| 2 | Dong (2025) | China  （Hunan） | 286 | Quantitative | DSES (Ulfert-Blank & Schmidt, 2022 | OLMS (Fowler, 2018) | A significant positive relationship was found between digital self-efficacy and online learning motivation (β = .432, p < .05). |
| 3 | Li et al., (2026) | China  (Sichuan) | 410 | Quantitative | Adapted Computer Self-Efficacy Scale (Compeau & Higgins, 1995) | Adapted Short Academic Motivation Scale (Kotera et al., 2023) | Self-efficacy had a significant positive effect on learning motivation (β = .278, p < .001). |
| 4 | Liang& Fan (2026) | China  (Sichuan) | 332 | Quantitative | Chinese version of the Regulatory Emotional Self-Efficacy Scale (RESE; Caprara et al.) | Learning Motivation Scale (LM; Huang Xiting; Li, 2022) | Regulatory emotional self-efficacy was significantly and positively correlated with learning motivation (r = .541, p < .001). |
| 5 | Lin et al., (2025) | Malaysia | 296 | Quantitative | English-Speaking Self-Efficacy Scale (Chen, 2009) | Learning Motivation Scale (Gao, 2003) | self-efficacy significantly and positively predicted English learning motivation (β = .24, p < .001). |
| 6 | Liu et al., (2026) | China  (Sichuan) | 519 | Quantitative | General Self-Efficacy Scale (GSES; Chen et al., 2001) | Revised Learning Process Questionnaire (R-LPQ; Kember et al., 2004) | Self-efficacy was significantly and positively correlated with learning motivation (r = .44, p < .001). |
| 7 | Shengyao et al., (2024) | China  (Zhejiang) | 518 | Quantitative | Self-Efficacy Scale (Rowbotham & Schmitz) | Academic Motivation Scale (Harter) | Self-efficacy had a significant positive effect on academic motivation (β = .642, p < .001). |
| 8 | Shi et al., (2026) | China  (Anhui) | 1484 | Quantitative | Chinese version of the Academic Self-Efficacy Scale (AS; Pintrich & De Groot, 1990; revised by Liang & Zhou, 2000) | Academic Motivation Scale (AMS; Biggs et al., 2001) | Academic self-efficacy was significantly and positively correlated with academic motivation (r = .486, p < .01). |
| 9 | Sindu et al., (2024) | Indonesia | 68 | Quantitative | Self-Efficacy Questionnaire (Bandura, 1994) | Motivated Strategies for Learning Questionnaire (MSLQ) | Self-efficacy significantly and positively predicted learning motivation (β = .428, p < .001). |
| 10 | Su et al., (2024) | China  (Guangdong) | 241 | Quantitative | Simplified General Self-Efficacy Scale (GSE; Schwarzer, 1997) | Adapted Academic Motivation Scale (AMS; Vallerand et al., 1992) | Self-efficacy significantly and positively predicted learning motivation (β = .176, p = .004). |
| 11 | Tang & Osman (2022) | China  (Shandong) | 1018 | Quantitative | Learning Self-Efficacy Scale (LSS; Liang, 2000) | Learning Motivation Scale (LMS; Tian & Pan, 2006) | Learning self-efficacy was significantly and positively correlated with learning motivation (r = .712, p < .01). |
| 12 | Zhang (2024) | China  (Sichuan) | 200 | Quantitative | Modified English Learning Self-Efficacy Scale (Zhang Risheng & Yuan Limin) | Adapted Learning Motivation Scale (Zhang Risheng & Yuan Limin; Lei Li et al.) | English learning self-efficacy significantly and positively predicted intrinsic motivation (β = .573, p < .001) and achievement motivation (β = .491, p < .05), but not extrinsic motivation (β = .054, p > .05). |

Table A2. Methodological Quality Assessment of the Included Studies

| **Study** | **Introduction** | **Background** | **Methods** | **Sampling** | **Data Collection** | **Ethical Matters** | **Results** | **Discussion** | **Total (40)** |
| --- | --- | --- | --- | --- | --- | --- | --- | --- | --- |
| Chen et al. (2026) | 5 | 4 | 4 | 3 | 4 | 5 | 5 | 5 | 35 |
| Dong (2025) | 5 | 4 | 5 | 3 | 5 | 5 | 5 | 4 | 36 |
| Li et al. (2026) | 5 | 5 | 5 | 5 | 5 | 4 | 5 | 4 | 38 |
| Liang & Fan (2026) | 5 | 5 | 5 | 3 | 4 | 5 | 5 | 5 | 37 |
| Lin et al. (2025) | 4 | 4 | 4 | 4 | 4 | 3 | 4 | 4 | 31 |
| Liu et al. (2026) | 4 | 4 | 4 | 3 | 4 | 5 | 4 | 4 | 32 |
| Shengyao et al. (2024) | 4 | 4 | 4 | 4 | 5 | 5 | 5 | 5 | 36 |
| Shi et al. (2026) | 5 | 5 | 5 | 3 | 5 | 5 | 5 | 5 | 38 |
| Sindu et al. (2024) | 4 | 4 | 4 | 3 | 4 | 4 | 4 | 4 | 29 |
| Su et al. (2024) | 5 | 5 | 5 | 4 | 5 | 4 | 5 | 5 | 38 |
| Tang & Osman (2022) | 4 | 4 | 4 | 4 | 4 | 3 | 4 | 4 | 30 |
| Zhang (2024) | 5 | 5 | 5 | 3 | 4 | 5 | 5 | 5 | 37 |
